# Emerging Trends of Beta-Lactamases in Western Nepal: A Growing Public Health Concern

**DOI:** 10.1155/ijm/3559197

**Published:** 2025-07-14

**Authors:** Rajan Paudel, Niranjan Nayak, Bipin Chapagain, Elina Shrestha, Deependra Hamal, Dharm Raj Bhatta, Bishnu Raj Tiwari

**Affiliations:** ^1^School of Health and Allied Sciences, Faculty of Health Sciences, Pokhara University, Pokhara, Nepal; ^2^Department of Microbiology, Manipal College of Medical Sciences, Pokhara, Nepal

**Keywords:** AmpC, carbapenemase, extended-spectrum *β*-lactamase

## Abstract

**Background:** Clinically challenging bacterial infections are caused by microorganisms producing extended-spectrum *β*-lactamases (ESBLs), AmpC *β*-lactamase (AmpC), and carbapenemases, which confer antibiotic resistance and may result in treatment failure. This study was aimed at determining the prevalence of ESBL, AmpC, and carbapenemase-producing clinical isolates of *Escherichia coli*, *Klebsiella pneumoniae*, and *Pseudomonas aeruginosa*.

**Methods:** This study was a cross-sectional study. A total of 362 isolates of *E. coli, K. pneumoniae*, and *P. aeruginosa* from urine, blood, pus, sputum, swab, and endotracheal (ET) tube tips were obtained from patients attending Manipal Teaching Hospital, Pokhara, Nepal, during March 2022 to October 2022. Phenotypic confirmation of ESBL, AmpC, and carbapenemase was done by combined disk test and modified Hodge test.

**Results:** The prevalence of ESBL among isolates was found to be 58.3%. Confirmative tests showed 65.3% *P. aeruginosa*, 30.2% *K. pneumoniae*, and 17.7% *E. coli* were AmpC producers. Among the carbapenem-resistant isolates, 81.7% showed phenotypic evidence of carbapenemase production based on the modified Hodge test. Additionally, 9.9% and 5.2% of isolates demonstrated phenotypic characteristics suggestive of metallo-*β*-lactamase (MBL) and *Klebsiella pneumoniae* carbapenemase (KPC) activity, respectively.

**Conclusion:** The ESBL production was the predominant mechanism of resistance to *β*-lactam drugs, followed by AmpC and carbapenemase production. Routine identification and monitoring of these organisms, followed by detection of *β*-lactamase production, optimize the effective management and prevention of antimicrobial resistance.

## 1. Background

Antibiotic resistance in Gram-negative bacterial pathogens is a global problem linked to high morbidity and mortality rates [1]. Multidrug resistance among Gram-negative pathogens related to Enterobacteriaceae, *Acinetobacter baumannii*, and *Pseudomonas aeruginosa* have risen alarmingly. The production of *β*-lactamase has been identified as a key mechanism for resistance to *β*-lactam antibiotics. Many distinct types of enzymes, such as extended-spectrum *β*-lactamases (ESBLs), AmpC *β*-lactamases, and carbapenemases, have been associated with resistance to various *β*-lactams [2, 3]. Organisms producing *β*-lactamases have developed multidrug resistance. The rising prevalence of bacterial strains producing multiple *β*-lactamases will create further complications [4]. ESBLs are enzymes that can break down monobactams, broad-spectrum cephalosporins, and penicillins [5]. ESBLs are frequently encoded by genes found on large plasmids, which has remarkable clinical significance [6]. In the 1980s, *Escherichia coli*, and *Klebsiella pneumoniae* generating ESBLs were first discovered, but nowadays, they are widely recognized [7]. AmpC *β*-lactamase belongs to the Class Molecular “C” of Ambler's classification. AmpC enzyme transfers the resistance genes to the penicillin group of drugs in addition to second- and third-generation cephalosporins [8]. AmpC genes or ESBL genes found on plasmids are passed from one bacterial strain to another, which is typically the reason for the emergence of third-generation cephalosporins resistance [6]. The CTX-M, TEM, and SHV kinds are the three main classes of ESBLs [5]. Reduced outer membrane permeability is accompanied by ESBLs and AmpC enzymes giving rise to resistance or reduce susceptibility. Furthermore, *β*-lactamase enzymes with carbapenemase activity are widely distributed throughout the world [9].

Many bacterial pathogens of the family Enterobacteriaceae, as well as a few other microorganisms, such as *Pseudomonas aeruginosa*, contain chromosomally encoded cephalosporinases called AmpC *β*-lactamases that cause resistance towards most penicillins, cefoxitin, cephalothin, cefazolin, and combinations of *β*-lactamase inhibitor with *β*-lactam antibiotics [10]. For enterobacterial pathogens that produce ESBL and AmpC enzymes, carbapenems have been regarded as the preferred treatment option. The presence of ESBL, AmpC, and carbapenemase enzymes is often associated with additional resistance mechanisms, such as reduced outer membrane permeability, further limiting the effectiveness of antibiotics and contributing to increased resistance or decreased susceptibility to carbapenems [9]. The Centers for Disease Control and Prevention (CDC) defines carbapenem-resistant Enterobacteriaceae (CRE) as those members of Enterobacteriaceae which are resistant to at least one carbapenem antibiotic (imipenem, meropenem, doripenem, or ertapenem) or those which produce carbapenemase enzymes [11].

Carbapenem resistance in Enterobacteriaceae can result from a variety of carbapenemases. The most frequent enzymatic cause of carbapenem resistance is the family of *Klebsiella pneumoniae* carbapenemase (KPC). KPC are serine-based Class A *β*-lactamases. Another significant group of carbapenemases is the New Delhi metallo-*β*-lactamase (MBL) (NDM) family of Class B MBLs. The NDM family of Class B MBLs contributes to carbapenem resistance by hydrolyzing carbapenem antibiotics. The acquisition of such carbapenemase genes by Gram-negative bacteria, including Enterobacterales, plays a significant role in the global emergence of carbapenem-resistant organisms [12, 13]. Infections due to carbapenemase-producing organisms have limited treatment options, which result in poor clinical outcomes. The carbapenemase enzymes VIM, IMP, NDM, KPC, and OXA are produced by several organisms [14]. Different mechanisms, including porin loss, excessive expression of efflux pumps or mutation, and different carbapenemase enzymes, result in resistance to carbapenems. Several genes, including *bla*_IMP_, *bla*_OXA-48-like_, *bla*_VIM_, *bla*_SIM_, *bla*_KPC_, and *bla*_NDM_, encode these enzymes [15]. This study determined the phenotypically proved ESBLs, AmpC, and carbapenemase-producing clinical isolates of *Escherichia coli*, *Klebsiella pneumoniae*, and *Pseudomonas aeruginosa*.

## 2. Materials and Methods

The cross-sectional study was carried out in the Department of Microbiology, Manipal Teaching Hospital (a 750-bedded tertiary care hospital), Pokhara, Nepal, from March 2022 to October 2022. A total of 362 isolates of *Escherichia coli*, *Klebsiella pneumoniae*, and *Pseudomonas aeruginosa* from different samples (urine, blood, pus, sputum, swab, and endotracheal (ET) tube tips) collected from in-patients and out-patients visiting Manipal Teaching Hospital were studied. Clinical isolates from patients of all age groups and gender were included. Isolation and identification of organisms were done by standard conventional microbiological techniques [16, 17]. The screening of probable ESBL, AmpC, and carbapenemase producers and their confirmation were done following Clinical and Laboratory Standards Institute (CLSI) guidelines [18] and other publications [19–22]. All antibiotic disks used for testing were purchased from HiMedia Laboratories Pvt. Ltd, Mumbai, India. Descriptive findings were analyzed in frequencies and percentages. Chi-square test was applied with cross tabulation. Statistical analysis was done by Microsoft Office Excel and IBM SPSS software Version 25.0. Statistical significance was determined if a *p* value of ≤ 0.05.

### 2.1. ESBL

#### 2.1.1. Screening for ESBL

CLSI guideline was followed for screening of ESBL by using cefotaxime (30 *μ*g) or ceftazidime (30 *μ*g) disks. Detection of ESBL was further examined in *E. coli* and *K. pneumoniae* isolates displaying inhibition zone of ≤ 22 mm for ceftazidime and of ≤ 27 mm, for cefotaxime, respectively. However ceftazidime (30 *μ*g) disk was taken for screening the isolates of *P. aeruginosa* for the production of ESBL.

#### 2.1.2. Phenotypic Confirmation of ESBL

The phenotypic confirmation of ESBL production was performed by combined disk test with ceftazidime (30 *μ*g) and ceftazidime/clavulanic acid (30/10 *μ*g) disks. The ceftazidime and ceftazidime with clavulanic acid disks were kept 20 mm apart. After overnight incubation at 37°C, an increase in inhibition zone diameter of ≥ 5 mm for ceftazidime in the presence of clavulanic acid in comparison to inhibition zone when tested ceftazidime alone was indicative of ESBL production [18].

### 2.2. AmpC *β*-Lactamase

#### 2.2.1. Screening of AmpC *β*-Lactamase

##### 2.2.1.1. Screening in *E. coli* and *K. pneumoniae*

Cefoxitin disk (30 *μ*g) was used for screening of *E. coli* and *K. pneumoniae* isolates that produced AmpC *β*-lactamase. An inhibitory zone of ≤ 18 mm towards cefoxitin disk was considered positive for AmpC. Such isolates were further tested for confirmation.

#### 2.2.2. AmpC *β*-Lactamase Confirmation

Phenotypic confirmation of AmpC production was done by using inhibition-based method for those isolates which were screened as AmpC producers. For this method, solution of phenylboronic acid (PBA) was prepared by dissolving 120 mg PBA in 3 mL of dimethyl sulfonic acid (DMSO), it was then filled with 3 mL distilled water. Twenty microliters of PBA stock solution was dispensed onto a cefoxitin (30 *μ*g) disk. The cefoxitin disk and cefoxitin disk with 400 *μ*g of PBA were kept on an MHA plate previously inoculated with the test isolate by following the standard disk diffusion technique. Disks were used right away after drying for 30 min. The inoculated plates were then kept in an incubator for 24 h at 37°C. An organism was considered to be an AmpC producer if the inhibitory zone towards cefoxitin disk increased by about ≥ 5 mm when PBA was added as compared to cefoxitin alone [19].

#### 2.2.3. AmpC *β*-Lactamase Production in *Pseudomonas aeruginosa*

##### 2.2.3.1. Screening for AmpC *β*-Lactamase Production

All the isolates that showed reduced susceptibility towards cefoxitin disk were suspected to produce AmpC, and those isolates needed phenotypic confirmation.

##### 2.2.3.2. Confirmation of AmpC *β*–Lactamase Production

Phenotypic confirmation of AmpC *β*-lactamase production in *P. aeruginosa* was conducted only for those isolates that had shown screening test positive according to a previously described methodology.

Ceftazidime (30 *μ*g) and cefotaxime (30 *μ*g) disks were spaced apart by 20 mm from the cefoxitin (30 *μ*g) disk on a MHA plate, which had already been inoculated with the *P. aeruginosa* test isolate, for the testing of AmpC *β*-lactamase synthesis. When the diameter of the zone of inhibition formed by any of the cephalosporins tested in combination with cefoxitin was ≥ 5 mm, the release of AmpC was identified. The designation of AmpC *β*-lactamase producers was also given to isolates that additionally displayed a blunting of the ceftazidime or cefotaxime inhibition zone close to the cefoxitin disk [20, 23].

### 2.3. Carbapenemase

#### 2.3.1. Screening of Carbapenemase

Imipenem and meropenem disks were used for screening of carbapenemase as per CLSI recommendations. The organisms resistant to any of these antibiotics were taken as possible carbapenemase producers. Carbapenemase production was confirmed phenotypically by the modified Hodge test (MHT).

#### 2.3.2. Confirmation of Carbapenemase by MHT

A 0.5 McFarland turbidity matched solution of *Escherichia coli* ATCC 25922 was prepared in 5 mL of saline or broth. After diluting 1:10, broth was lawned on a plate of the Mueller–Hinton agar.

A meropenem or imipenem (10 *μ*g) disk was placed in the center of the plate. The streaking of a clinical isolate was made in a straight line away from the border of a disk to the plate edge. For 16–24 h, the plate was incubated at 35 ± 2°C.

*Escherichia coli* 25922, demonstrating a clover leaf-like indentation along the diffusion zone, along the growth line of the test organism after 24 h, was classified as MHT positive. If test isolate showed inhibition of growth of *Escherichia coli* 25922 along with streak of test-organism growth inside the disk diffusion, the test was considered as negative for MHT [21, 22].

### 2.4. MBL

Phenotypic detection of an MBL was done by combined-disk test in a single agar plate. For this test one imipenem (IPM) disk (10 *μ*g) and another IPM plus 10 *μ*l anhydrous EDTA of 0.1 M (292 *μ*g) had been spaced 25 mm apart. The zone of inhibition around the imipenem and imipenem with EDTA was compared following 16–18 h of aerobic incubation at 37°C.

Inhibition zone around the IPM-EDTA disk increased by about ≥ 5 mm in comparison to imipenem disk alone was indicated as an MBL positive [24].

### 2.5. KPC

Isolates which were detected as carbapenem resistant were further processed for phenotypic confirmation of KPC with the meropenem PBA disk diffusion test. The increase in inhibition zone around the meropenem disk with PBA of ≥ 5 mm in contrast to meropenem alone was considered a presumptive KPC producer [25].

### 2.6. Differentiation and Coproduction of Carbapenemases (MBLs and Class A KPC)

The distinction of KPC and MBL enzymes and the phenotypic identification of carbapenemase synthesis was performed using a combined disk test with the help of meropenem and meropenem with 400 *μ*g of PBA or 292 *μ*g of EDTA or 292 *μ*g of EDTA and 400 *μ*g of PBA both.

Production of KPC was considered to be positive if the diameter of zone of inhibition around the meropenem having PBA and EDTA both increased by ≥ 5 mm in comparison with diameter of the zone of inhibition seen towards meropenem disk alone.

Production of MBL was taken positive if the zone of inhibition around the meropenem disk with EDTA and PBA both changed by ≥ 5 mm in comparison to the diameter of the zone of inhibition of the meropenem disk alone.

Both KPC and MBL enzyme productions were considered positive when the inhibition zone diameter around the meropenem disk supplemented with PBA (for KPC) or EDTA (for MBL) showed a ≥ 5 mm increase compared to the meropenem disk alone. Isolates demonstrating a < 5 mm increase in zone diameter with either of the inhibitors (meropenem + PBA or meropenem + EDTA) were classified as negative for the production of the respective enzyme.

If none of these three combined disk tests followed above criteria, the isolate was then phenotypically indicated negative for the production of KPC as well as MBL [25, 26].

## 3. Results

A total of 362 isolates of *E. coli*, *K. pneumoniae*, and *P. aeruginosa* were obtained. Patients belonged to the age group of 12 days to 96 years. The mean age was 50.37 years (mean ± SD = 50.37 ± 23.39). The maximum number of patients was from the age group 61–80 years. The lowest number of patients belonged to the age group 81 years and above. Out of 362 patients, 186 (51.4%) were males, and 176 (48.6%) were females. The maximum number of samples, that is, 153 (42.3%) were from patients visiting different outpatient departments (OPDs) of the hospital, followed by different wards, 130 (35.9%), ICUs, 43 (11.9%), and emergency, 36 (9.9%). The majority of isolates were from urine samples, accounting for 223 (61.6%). Besides, there were 17 (4.7%) isolates from blood, 8 (2.2%) from ET tube tips, 31 (8.6%) from pus, 67(18.5%) from sputum samples, and 16 (4.4%) from swabs. Among 362 isolates, the maximum number of isolates were *E. coli*, followed by *K. pneumoniae* and *P. aeruginosa*. The total isolates of *E. coli* were 158 (43.6%), *K. pneumoniae* were 106 (29.3%), and *P. aeruginosa* were 98 (27.1%) as shown in Table 1.

The screening test for ESBL, AmpC, and carbapenemase was done by disk diffusion method. The overall prevalence of ESBL production among isolates was noted to be 58.3% (211 of 362). Phenotypic confirmative analysis showed that 65.3% *P. aeruginosa*, 30.2% *K. pneumoniae*, and 17.7% *E. coli* were AmpC producers. The MHT was done in 60 isolates having reduced susceptibility towards carbapenem antibiotics. A total of 49 (81.7%) carbapenem-resistant isolates were found positive by MHT (Table 2).

*E. coli* and *K. pneumoniae* mostly produced MBL, whereas the majority of carbapenem-resistant isolates of *P. aeruginosa* produced Class A KPC. 0.6% of the total isolates were found producing both KPC and MBL. Among carbapenem-resistant isolates, 1.9% were found negative for both KPC and MBL. The overall prevalence of MBL was 9.9%, and KPC was 5.2% (Table 3).

Table 4 indicated that the maximum numbers of ESBL-producing isolates were obtained from patients visiting OPDs, whereas maximum numbers of AmpC, carbapenemase, MBL, and KPC producing isolates were detected from patients admitted to different wards of the hospital. Isolates from patients visiting different units of hospital were found to be significantly associated with the production of ESBL, AmpC, carbapenemase, MBL, and KPC. There was no significant association between age groups and different types of *β*-lactamases (Table 4).

## 4. Discussion

The increasing rate of multidrug resistance among the bacterial pathogens has been a serious issue globally. Multidrug-resistant Gram-negative bacterial pathogens have been significantly associated with community and hospital acquired infections. Gram-negative bacterial antimicrobial resistance is rising primarily as a result of the spread of strains that produce ESBLs, AmpC *β*-lactamases, and carbapenemases [27].

This study was carried out to assess the ESBL, AmpC, and carbapenemase production in *E. coli*, *K. pneumoniae*, and *P. aeruginosa* clinical isolates. In the present study, the highest number of growths of these pathogenic strains was seen among patients within the age group 61-80 years. This may be due to the fact that patients belonging to this age group might be more vulnerable to infection. The majority of patients were males, 186 (51.4%) as compared to females, 176 (48.6%). The number of isolates was greater from patients visiting OPDs, followed by different wards, ICU, and emergency, which may vary according to the number of patients visiting in each department and the collected sample numbers. *E. coli* was the most common organism reported from the patients visiting OPDs, followed by *K. pneumoniae* and *P. aeruginosa*. As *E. coli* and *K. pneumoniae* are the commonest organisms reported in the clinical specimens [28, 29], *K. pneumoniae* was found predominant among patients admitted in ICU.

In the present study, 63.9% of the total *E. coli*, 49.1% of *K. pneumoniae*, and 59.2% isolates of *P. aeruginosa* were confirmed as ESBL producers. The overall prevalence of ESBL production was 58.3%. This rate is higher than other studies reported from Nepal: Guragain et al., 29.8%; Neupane et al., 33.2%; Kayastha et al., 28.2%; Shrestha et al., 18%; and Raut et al., 22.4% [30–34]. The ESBL production was similar with the findings of Manandhar et al. [35] and Surgers et al. [36]. The variation of ESBL production may be due to plasmid mediated transfer between isolates, excessive use of the cephalosporins, and severity of the illness [37, 38].

Additionally, our findings revealed a comparatively less occurrence of ESBL in *E. coli* as compared with study of Govindaswamy et al. [39] from India, who reported 88.3% were ESBL producers, and another study from India also reported a very high prevalence among urinary isolates, that is, 92.2% positive for ESBL [40]. Comparatively less ESBL-producing isolates of *E. coli* are also reported by Nisha et al., that is, 37.5% from India, and Masud et al. reported that 40.9% uropathogens were ESBL producer from Bangladesh [41, 42]. The use of broad spectrum antibiotics, prescribing habit and various antibiotic use policies in various countries, healthcare settings, and institutions may be responsible for this diversity of ESBL producing strains [38, 43].

The AmpC production was detected in 17.7% *E. coli*, 30.2% *K. pneumoniae*, which is slightly higher than the rate of AmpC production reported by Kazemian et al. [3]. The prevalence of AmpC in *P. aeruginosa* was 65.3%, which is much higher than the findings of Goel et al. [44]. The AmpC overexpression and resistance to *β*-lactam antibiotics in *P. aeruginosa* may be mediated by chromosomally encoded *β*-lactamases, plasmid-mediated *β*-lactamases, outer membrane porin deletions, point mutations in AmpC, and the overexpression of the efflux pump [45].

In the current study, majority of AmpC producing isolates were isolated from patients visiting different wards of the hospital. The increasing prevalence of AmpC *β*-lactamase production in Gram-negative bacterial isolates from both the community and hospital settings is quickly becoming a global issue. Since the presence of an AmpC *β*-lactamase frequently appears to result in treatment failure, which also creates a challenge in available treatment options, another study conducted in India reported that AmpC *β*-lactamase was found in 59.4% of *P. aeruginosa* isolates, indicating a almost similar prevalence of AmpC producer in respect to the present study, but the study conducted in Nigeria showed that 36% of *P. aeruginosa* isolates have AmpC *β*-lactamase production, which was in contrast with the findings of this study [20, 23].

In the present study, majority of ESBLs and Ampc *β*-lactamases produced by GNB were detected in urine samples, and isolates from adult patients 61–80 years of age had a higher ESBL proportion, which is similar with the study done in Addis Ababa, Ethiopia [46]. Shashwati et al. [47] also found that the greatest number of ESBL-producing isolates was reported from patient urine samples. The possible reason for similar findings might be because of greater numbers of urine samples that were taken in this study and the number of patients belonging to 61–80 years age.

Carbapenems are one of the classes of *β* lactam antibiotics used as the antibiotics as the last resort for the treatment of bacterial infections due to Gram-negative bacilli. In this study, 81.7% of the carbapenem resistant isolates were confirmed as carbapenemase producers by MHT. Similar finding (82%), by applying MHT, was reported by Shanmugam et al., in a study done in Chennai, India [48]. Carbapenem resistance among bacteria may be due to a variety of factors including overexpression of efflux pump, *β*-lactamase enzyme mediated resistance, plasmid mediated transfer of resistance, and structural mutaions [49].

In this study, out of total isolates exhibiting carbapenem resistance, 19 were Class A KPC producers, and 36 were Class B MBL producers. The overall prevalence of KPC and MBL was 5.2% and 9.9%, respectively. This finding is in contrast with the studies reported by Dhungana et al. [50] and Kuinkel et al. [37] from Kathmandu, Nepal. This might be due to sample size and variation of the study subjects.

Study done by Bora et al. [51] in Central Nepal reported the comparatively higher prevalence of MBL as compared to our study. In their study, prevalence of MBL in *E. coli* was 18.98%, and in *K. pneumoniae*, it was 21.08% among different clinical samples, but in this study, the prevalence of MBL in *E. coli* and *K. pneumoniae* was 8.2% and 16.0%, respectively. However, only 4% isolates of *E. coli* and *K. pneumoniae* were reported as MBL producers by Nepal et al., in Kathmandu, Nepal [28]. In the present study, the prevalence of MBL in *P. aeruginosa* was found 6.1% (6/98). However, a very high prevalence, 24.4% (24/98) of MBL production in *P. aeruginosa*, was reported by Acharya et al. [52], which is study done in a tertiary care hospital of Kathmandu, Nepal, whereas another study by Baniya et al. [53] in Kathmandu reported that 16.4% isolates of *P. aeruginosa* were MBL producers.

Class A KPC enzymes and Class B MBLs are occurring more frequently, so it is important to differentiate between these enzymes in order to treat patients effectively and prevent treatment failure [25].

In the present study, 0.6% *E. coli*, 5.7% *K. pneumoniae*, and 12.2% *P. aeruginosa* were found to be Class A KPC producers. These findings of *E. coli* were in contrast with the findings of Dhungana et al. [50] who reported a KPC production among isolates of *E. coli* to be 14.4% and were in line with the findings of *K. pneumoniae*, that is, 7.1%.

## 5. Conclusion

The overall prevalence of ESBL production among isolates was found to be 58.3%. AmpC production was phenotypically confirmed in 65.3% of *P. aeruginosa*, 30.2% of *K. pneumoniae*, and 17.7% of *E. coli* isolates. The highest number of carbapenemase producers was found in *K. pneumoniae* followed by *P. aeruginosa* and *E. coli*. A total of 81.7% carbapenem resistant isolates were MHT positive. The prevalence of KPC in *E. coli*, *K. pneumoniae*, and *P. aeruginosa* was 0.6%, 5.7%, and 12.2%, respectively. Similarly, the prevalence of MBL in *E. coli* was 8.2%, in *K. pneumoniae* 16.0% and in *P. aeruginosa* 6.1%. The overall prevalence of KPC was 5.2%, and MBL was 9.9%. The major resistance mechanism behind carbapenem antibiotic resistance was the production of the carbapenemase enzyme. Laboratory performing antibiotic sensitivity test should be encouraged to identify ESBL, AmpC, and carbapenemase-producing organisms early, as this is important for preventing their spread and ensuring proper treatment.

## Figures and Tables

**Table 1:** Distribution of the isolates in accordance with the patient demographics, nature of samples, and treatment location (source of sample collection).

	***E. coli* (** **n** = 158**)**	***K. pneumoniae* (** **n** = 106**)**	***P. aeruginosa* (** **n** = 98**)**
	**Number (%)**	**Number (%)**	**Number (%)**
*Age*			
≤ 20 years	13 (8.2)	14 (13.2)	14 (14.3)
21–40 years	54 (34.2)	19 (17.9)	23 (23.5)
41–60 years	27 (17.1)	20 (18.9)	19 (19.4)
61–80 years	53 (33.5)	46 (43.4)	38 (38.7)
≥ 81 years	11 (7.0)	7 (6.6)	4 (4.1)
*Sex*			
Female	105 (66.5)	40 (37.7)	31 (31.6)
Male	53 (33.5)	66 (62.3)	67 (68.4)
*Source*			
Emergency	18 (11.4)	7 (6.6)	11 (11.2)
ICU	10 (6.3)	21 (19.8)	12 (12.3)
OPD	80 (50.6)	43 (40.6)	30 (30.6)
Ward	50 (31.7)	35 (33.0)	45 (45.9)
*Sample type*			
Blood	4 (2.5)	4 (3.8)	9 (9.2)
ET tube tips	0 (0.0)	3 (2.8)	5 (5.1)
Pus	9 (5.7)	14 (13.2)	8 (8.2)
Sputum	8 (5.1)	24 (22.7)	35 (35.7)
Swab	6 (3.8)	5 (4.7)	5 (5.1)
Urine	131 (82.9)	56 (52.8)	36 (36.7)

**Table 2:** Screening and confirmation of ESBL, AmpC, and carbapenemase among different isolates.

**Organisms**	**ESBL screening**		**ESBL confirmation**	
	**Negative**	**Positive**	**Negative**	**Positive**
	**N** ** (%)**	**N** ** (%)**	**N** ** (%)**	**N** ** (%)**

*E. coli*	47 (29.7)	111 (70.3)	10 (6.3)	101 (63.9)
*K. pneumoniae*	36 (34.0)	70 (66.0)	18 (17.0)	52 (49.0)
*P. aeruginosa*	34 (34.7)	64 (65.3)	6 (6.1)	58 (59.2)

**Organisms**	**AmpC screening**		**AmpC confirmation**	
	**Negative**	**Positive**	**Negative**	**Positive**
	**N** ** (%)**	**N** ** (%)**	**N** ** (%)**	**N** ** (%)**

*E. coli*	126 (79.7)	32 (20.3)	4 (2.5)	28 (17.7)
*K. pneumoniae*	66 (62.3)	40 (37.7)	8 (7.5)	32 (30.2)
*P. aeruginosa*	14 (14.3)	84 (85.7)	20 (20.4)	64 (65.3)

**Organisms**	**Carbapenemase screening**		**Carbapenemase confirmation**	
	**Negative**	**Positive**	**Negative**	**Positive**
	**N** ** (%)**	**N** ** (%)**	**N** ** (%)**	**N** ** (%)**

*E. coli*	145 (91.8)	13 (8.2)	3 (1.9)	10 (6.3)
*K. pneumoniae*	77 (72.6)	29 (27.4)	7 (6.6)	22 (20.8)
*P. aeruginosa*	80 (81.6)	18 (18.4)	1 (1.0)	17 (17.3)

**Table 3:** Differentiation of carbapenem resistant isolates.

**Organisms**	**Carbapenem resistant isolates**			
	**Class A, KPC**	**Class B, MBL**	**Both KPC and MBL**	**Neither KPC nor MBL**
	**N** ** (%)**	**N** ** (%)**	**N** ** (%)**	**N** ** (%)**
*E. coli*	1 (0.6)	13 (8.2)	1 (0.6)	0 (0)
*K. pneumoniae*	6 (5.7)	17 (16.0)	1 (0.9)	7 (6.6)
*P. aeruginosa*	12 (12.2)	6 (6.1)	0 (0)	0 (0)
Total	19 (5.2)	36 (9.9)	2 (0.6)	7 (1.9)

**Table 4:** Prevalence of ESBL, AmpC, carbapenemase, MBL, and KPC producer isolates (*n* = 362).

	**ESBL**	**AmpC**	**Carbapenemase**	**MBL**	**KPC**
	**N** ** (%)**	**N** ** (%)**	**N** ** (%)**	**N** ** (%)**	**N** ** (%)**
*Age*					
≤ 20 years	23 (10.9)	16 (12.9)	9 (18.4)	8 (22.2)	1 (5.3)
21–40 years	56 (26.6)	31 (25.0)	10 (20.4)	7 (19.4)	5 (26.3)
41–60 years	39 (18.5)	22 (17.7)	8 (16.3)	6 (16.7)	3 (15.8)
61–80 years	79 (37.4)	46 (37.1)	18 (36.7)	12 (33.4)	8 (42.1)
≥ 81 years	14 (6.6)	9 (7.3)	4 (8.2)	3 (8.3)	2 (10.5)
*p* value	0.396	0.964	0.596	0.473	0.712
*Sex*					
Female	96 (45.5)	45 (36.3)	19 (38.8)	14 (38.9)	8 (42.1)
Male	115 (54.5)	79 (63.7)	30 (61.2)	22 (61.1)	11 (57.9)
*p* value	0.184	< 0.001⁣^∗^	0.032⁣^∗^	0.075	0.052
*Source*					
Emergency	20 (9.5)	9 (7.3)	4 (8.2)	1 (2.8)	3 (15.8)
ICU	26 (12.3)	17 (13.7)	7 (14.3)	4 (11.1)	2 (10.5)
OPD	83 (39.3)	34 (27.4)	15 (30.6)	9 (25.0)	7 (36.8)
Ward	82 (38.9)	64 (51.6)	23 (46.9)	22 (61.1)	7 (36.8)
p value	0.038⁣^∗^	< 0.001⁣^∗^	0.031⁣^∗^	0.019⁣^∗^	0.016⁣^∗^
*Sample type*					
Blood	9 (4.3)	5 (4.0)	1 (2.0)	0 (0)	2 (10.5)
ET tube tips	2 (0.9)	5 (4.0)	2 (4.1)	1 (2.8)	0 (0)
Pus	21 (10.0)	9 (7.3)	6 (12.2)	6 (16.7)	0 (0)
Sputum	33 (15.6)	34 (27.4)	8 (16.3)	3 (8.3)	2 (10.5)
Swab	11 (5.2)	5 (4.0)	0 (0)	1 (2.8)	0 (0)
Urine	135 (64.0)	66 (53.2)	32 (65.3)	25 (69.4)	15 (78.9)
p value	0.004⁣^∗^	< 0.001⁣^∗^	0.413	0.095	0.110
*Organisms*					
*E. coli*	101 (47.9)	28 (22.6)	10 (20.4)	13 (36.1)	1 (5.3)
*K. pneumoniae*	52 (24.6)	32 (25.8)	22 (44.9)	17 (47.2)	6 (31.6)
*P. aeruginosa*	58 (27.5)	64 (51.6)	17 (34.7)	6 (16.7)	12 (63.2)
*p* value	0.009⁣^∗^	< 0.001⁣^∗^	< 0.001⁣^∗^	< 0.001⁣^∗^	< 0.001⁣^∗^

⁣^∗^Statistically significant at *p* < 0.05.

## Data Availability

The data that support the findings of this study are available from the corresponding author upon reasonable request.
